# Acute Moderate Hypoxia Reduces One-Legged Cycling Performance Despite Compensatory Increase in Peak Cardiac Output: A Pilot Study

**DOI:** 10.3390/ijerph18073732

**Published:** 2021-04-02

**Authors:** Hannes Gatterer, Verena Menz, Martin Burtscher

**Affiliations:** 1 Institute of Mountain Emergency Medicine, Eurac Research, 39100 Bolzano, Italy; 2 Department of Sport Science, University of Innsbruck, 6020 Innsbruck, Austria; verena.menz@uibk.ac.at (V.M.); Martin.Burtscher@uibk.ac.at (M.B.); 3 Austrian Society for High Altitude and Mountain Medicine, 6020 Innsbruck, Austria

**Keywords:** moderate altitude, small muscle mass, exercise performance, single-leg exercise

## Abstract

In severe hypoxia, single-leg peak oxygen uptake (VO_2peak_) is reduced mainly due to the inability to increase cardiac output (CO). Whether moderate altitude allows CO to increase during single-leg cycling, thereby restoring VO_2peak_, has not been extensively investigated. Five healthy subjects performed an incremental, maximal, two-legged cycle ergometer test, and on separate days a maximal incremental one-leg cycling test in normoxia and in moderate hypoxia (fraction of inspired oxygen (FiO_2_) = 15%). Oxygen uptake, heart rate, blood pressure responses, power output, and CO (PhysioFlow) were measured during all tests. Moderate hypoxia lowered single-leg peak power output (154 ± 31 vs. 128 ± 26 watts, *p* = 0.03) and oxygen uptake (VO_2_) (36.8 ± 6.6 vs. 33.9 ± 6.9 mL/min/kg, *p* = 0.04), despite higher peak CO (16.83 ± 3.10 vs. 18.96 ± 3.59 L/min, *p* = 0.04) and systemic oxygen (O_2_) delivery (3.37 ± 0.84 vs. 3.47 ± 0.89 L/min, *p* = 0.04) in hypoxia compared to normoxia. Arterial–venous O_2_ difference (a–vDO_2_) was lower in hypoxia (137 ± 21 vs. 112 ± 19 mL/l, *p* = 0.03). The increases in peak CO from normoxia to hypoxia were negatively correlated with changes in mean arterial pressure (MABP) (*p* < 0.05). These preliminary data indicate that the rise in CO was not sufficient to prevent single-leg performance loss at moderate altitude and that enhanced baroreceptor activity might limit CO increases in acute hypoxia, likely by reducing sympathetic activation. Since the systemic O_2_ delivery was enhanced and the calculated a–vDO_2_ reduced in moderate hypoxia, a potential diffusion limitation cannot be excluded.

## 1. Introduction

During exercise involving a large muscle mass (e.g., two-leg cycling), maximal oxygen uptake (VO_2max_) and exercise performance are mainly limited by cardiac output (CO) and thus the ability to deliver oxygen (O_2_) to the working muscles. Besides CO and the oxygen content of the arterial blood (CaO_2_), blood flow to and O_2_ extraction of contracting skeletal muscles are also determinants of VO_2max_ [1,2,3,4]. On the contrary, when exercising with a small muscle mass (e.g., single-leg cycling, arm or knee-extensor exercise), CO is not considered the limiting factor for VO_2max_ attainment, since maximal CO values are generally not achieved [5,6,7].

In acute hypoxia (e.g., high altitude) conditions, CaO_2_ and arterial O_2_ partial pressure (PaO_2_), which constitute the driving pressure behind O_2_ diffusion [8], are reduced. Thus, if not compensated by an increased CO and/or O_2_ extraction, aerobic capacity may be impaired, even when exercising with a small muscle mass. Indeed, in circumstances of severe hypoxia, similar or reduced CO and leg blood flow during single-leg exercise have been observed compared to normoxic conditions, which translates into reduced leg O_2_ delivery [1,9] and impaired VO_2max_. Thus, CO, despite ample functional reserve, seems not to be sufficiently increased to compensate for the reduced CaO_2_ in acute severe hypoxia [1]. In this regard, it is important to mention that increasing CO would not only increase leg O_2_ delivery, but also shorten capillary transit time and may thus limit time for O_2_ diffusion [5,7,10]. O_2_ extraction demonstrates a marked plateau in hypoxia and maximal values are similar in normoxia, indicating that muscle diffusive capacity plays a role in the VO_2max_ reduction at altitude [9,11]. The magnitude of this diffusion limitation is, however, controversially discussed [8,12,13]. Due to restoration of CaO_2_ during altitude acclimatization and single-leg VO_2peak_, O_2_ delivery, and not diffusional limitation at the muscle level, has been suggested as the primary reason for the VO_2peak_ reduction in acute hypoxia [1].

It needs to be considered that the study design, the actual muscle mass involved, as well as the participant’s fitness level may influence the physiological responses during exercise performed in hypoxia [6,9,12]. Rowell et al. [5], for instance, investigated untrained participants performing single-leg knee extension exercise at a peak workload stage, which was determined by pre-testing. Using this study design, CO and muscle blood flow reached higher values in severe hypoxia compared to normoxia [5], which is in contrast to the findings reported for well-trained athletes performing graded exercise tests to exhaustion [1,9,11]. 

Besides exercise setting and physical fitness levels of participants, the severity of hypoxia (altitude level) may also play a critical role when assessing factors limiting VO_2peak_ of small muscle mass in hypoxia [14]. The aforementioned studies performed exercise testing in severe hypoxia (FiO_2_ < 0.12 corresponding to altitude levels > 4000 m), with a large impact on CaO_2_ and O_2_ delivery to the myocardium, respiratory muscles, and the central nervous system (CNS) [1]. Low brain O_2_ delivery thus could have caused a cardioinhibitory reflex, eventually preventing a compensatory increase in CO [1]. Interestingly, to the best of our knowledge, only limited information is available on whether similar effects may occur in moderate hypoxia (i.e., FiO_2_ = 15%), where PaO_2_ and CaO_2_ are less affected. Physical activity involving small muscle mass is commonly performed at moderate altitudes, e.g., by workers, tourists, and athletes. Thus, insights on a potential loss of small muscle performance and related mechanisms when acutely exposed to rather moderate altitude is of scientific, as well as clinical/practical, relevance. 

To investigate whether moderate hypoxia allows CO to increase during exercise with a small muscle mass, eventually restoring normoxic O_2_ delivery and VO_2peak_, we evaluated hemodynamic and performance parameters during maximal single-leg exercise in normoxia and at a FiO_2_ of 15%. Even though muscle O_2_ diffusion might be affected by a reduced transit time, we hypothesized that at a moderate altitude, small muscle performance would be preserved by compensatory increases in CO and oxygen delivery.

## 2. Materials and Methods

### 2.1. Participants

Five healthy and fit subjects (three males, age: 41 ± 13 years; two females, age: 45 ± 7 years) volunteered to participate in this pilot study. Inclusion criteria were age between 25 and 55 years, no recent health problems (checked by routine medical examination) and no exposures to hypoxia or altitude over 1500 m at least 4 weeks before exercise testing. All participants regularly participated (at least 1 h per day on 3 days per week) in various sports (e.g., running, cycling, swimming) and did not complete specific cycling training prior to their participation. Their baseline characteristics are shown in Table 1. Subjects gave their informed consent prior to their inclusion in the study, which has been performed in accordance with the ethical standards laid down in the Declaration of Helsinki. The study was approved by the local ethics review board (University of Innsbruck, Department of Sport Science, ZI.014/2010).

### 2.2. Protocol

**Figure 1:**
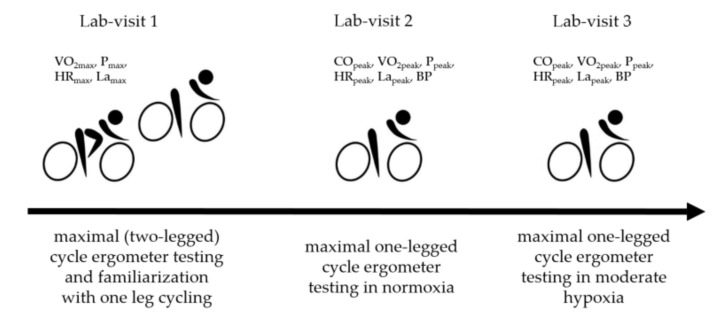
Experimental design. Blood pressure, BP; cardiac output, CO; heart rate, HR; lactate concentration, La; oxygen uptake, VO_2_; power output, P.

Participants visited the laboratory on three separate days: day 1 for routine medical examination, maximal (two-legged) cycle ergometer testing, and familiarization with one-leg cycling; days 2 and 3 for maximal one-legged (dominant leg) cycling tests in normoxia and hypoxia (Figure 1).

### 2.3. One-Legged Exercise Tests

The one-legged cycle ergometer tests were conducted on an electrodynamically braked cycle ergometer (Excalibur Sport, Lode, the Netherlands). Both trials were performed in the late morning on separate days after a light breakfast at least 2 h prior to the test and a 10 min warm-up period (two-legged cycling at 40 watts). The handlebar and the saddle of the cycle ergometer were individually adjusted and remained the same in both trials. Resting measurements were performed in a sitting position (10 min) on the cycle ergometer. After this, the test started with a workload of 20 watts, which was increased by 20 watts every 2 min until the subject was unable to maintain a pedaling frequency of 30 rpm. The first test was performed in normoxia (FiO_2_ = 20.9%) and the second in hypoxia (FiO_2_ = 15%). Subjects were blinded to the applied FiO_2_. Hypoxic air was delivered by the Hypoxico Altitude Generator (Hypoxico Europe GmbH) via a rigid mouthpiece connected to a “Y” system fixation with a double valve ensuring separate pathways between inspired and expired flow. The same valve system was applied during the normoxia setting.

### 2.4. Measurements and Calculation

Expired gases were analyzed for the assessment of peak oxygen uptake (VO_2peak_) by a gas analyzer (Oxycon Alpha, Jaeger, Germany); 15 s averages of peak values were taken for analysis. Peripheral oxygen saturation (SpO_2_) was continuously measured by finger pulse oximetry (Nonin, Sanesco, Austria). Blood was taken from the hyperemized earlobe at rest and shortly before finishing the exercise test for determination of PaO_2_ and hemoglobin concentration (ABL 80 Flex CO-OX OSM, Radiometer, Kopenhagen, Denmark). Lactate concentration (La) was determined at rest before starting the exercise test and 3 min after test termination (Biosen C-line, EKF diagnostic, Barleben, Germany). For continuous measurement of hemodynamic parameters (heart rate (HR), stroke volume (SV), CO, and total peripheral resistance (TPR)), the non-invasive impedance cardiograph PhysioFlow (Manatec biomedical, Poissy, France) was applied. Six electrodes were properly placed on the neck and chest, according to the manufacturer’s instructions. Arterial blood pressure (BP) was measured at rest (sitting on the cycle ergometer) for calibration of the PhysioFlow before each exercise test and at the end of each stage. The signal quality and stability were checked over the entire test period.

Arterial–venous O_2_ difference (a–vDO_2_, CaO_2_ − CvO_2_) was calculated using the Fick equation a–vDO_2_ = VO_2_/CO [15]. Arterial oxygen content (CaO_2_) was calculated as: (Hb × 1.34 × SaO_2_) + (PaO_2_ × 0.003), and systemic O_2_ delivery as: CO × CaO_2_. Mean arterial blood pressure (MABP) was calculated as: 1/3 (systolic − diastolic) + diastolic BP, and total peripheral resistance (TPR) as: MABP/CO.

### 2.5. Statistics

Data are presented as mean values (±standard deviation, SD). Wilcoxon signed-rank tests were used to compare means recorded in normoxia and hypoxia. Spearman correlation analyses were performed to test associations between two variables. *p*-values < 0.05 are considered to indicate statistical significance. 

## 3. Results

Resting SpO_2_ values were higher in normoxia compared to hypoxia, whereas a lower resting HR was observed in normoxia (Table 2).

**Figure 2:**
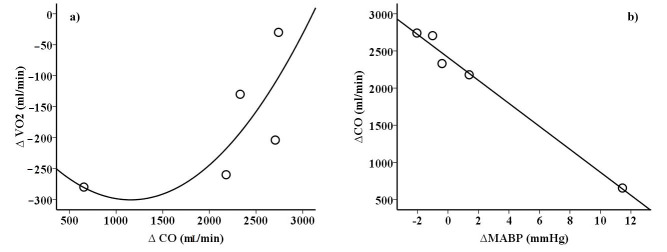
Relationship between changes from normoxia to hypoxia of (**a**) peak oxygen consumption (VO_2_) and cardiac output (CO) (r^2^ = 0.91, Spearman) and (**b**) CO and mean arterial blood pressure (MABP) (r^2^ = 1.0, Spearman).

Resting BP did not differ between hypoxia and normoxia (124/76 vs. 122/74 mmHg *p* > 0.05, respectively). Peak one-legged cycling values in normoxia and hypoxia are illustrated in Table 3. During maximal single-legged exercise, peak CO was higher in hypoxia compared to normoxia, mainly due to an elevated peak HR. CaO_2_ was reduced at simulated altitude (lower SpO_2_ and PaO_2_ and unaffected Hb). The higher CO, despite the reduced CaO_2_, resulted in a higher systemic O_2_ delivery in hypoxia compared to normoxia. Nonetheless, peak power output (*p* = 0.03, effect size (Cohen’s d) = 4.8) and peak VO_2_ (*p* = 0.04, effect size (Cohen’s d) = 1.0) were lower in hypoxia compared to normoxia. In line with this, a–vDO_2_ was lower in hypoxia. Peak La concentration was higher in hypoxia compared to normoxia. Differences in peak CO between the normoxia and hypoxia sessions were positively correlated with differences in peak VO_2_ (r^2^ = 0.91, *p* < 0.05) (Figure 2).

Changes (increase) of peak CO values from normoxia to hypoxia were negatively correlated with MABP in normoxia (r^2^ = 0.91, *p* < 0.05), MABP in hypoxia (r^2^ = 0.91, *p* < 0.05), and MABP changes (r^2^ = 1.0, *p* < 0.05, Figure 2).

## 4. Discussion

The main results of the present study show reduced peak power output (P_peak_) and VO_2peak_ values despite elevated systemic oxygen delivery during one-legged cycling in acute moderate hypoxia compared to normoxia. Data indicate that the CO increase was not sufficient to prevent the decline in P_peak_ and VO_2peak_ in hypoxia, and that the individual magnitude of VO_2peak_ reduction was closely associated with individual CO responses. This contrasts with our hypothesis, as we predicted unchanged one-leg performance due to compensation of the lower FiO_2_ and SpO_2_ by increased CO, eventually supported by hemoconcentration. Since systemic O_2_ delivery was enhanced and a–vDO_2_ reduced in moderate hypoxia compared to normoxia, a potential diffusion limitation cannot be excluded.

P_peak_ and VO_2peak_ during two-legged cycling (large muscle mass involved) are primarily limited by CO and the associated ability to deliver O_2_ to the working muscles [1,16,17]. This, however, does not apply when exercising with a small muscle mass, i.e., one-legged cycling, because maximal CO in general is not attained [5,6,7]. In the present study, peak values of CO and VO_2_ are relatively (in proportion to the involved muscle mass) higher during cycling with one compared to two legs, i.e., 75% of two-legged VO_2peak_. Theoretically, there is still compensatory potential for CO to increase when oxygen delivery during cycling with one leg is affected by hypoxia, i.e., oxygen desaturation, which was actually confirmed by our findings. Why compensation is insufficient to restore performance and varies considerably between individuals remains elusive. 

A novel finding of the present study, besides showing a partial compensatory increase in peak CO at moderate altitude, is the observation that MABP and CO changes from normoxia to hypoxia correlate closely (r = −0.91 to −1.0). Although this result appears to be influenced by a single subject displaying a large increase in MABP, it seems intriguing to speculate that baroreceptor activity might be involved in the regulation of CO in acute moderate hypoxia (Figure 2). Baroreflex sensitivity (hypocapnia triggered) is usually reduced in acute hypoxia, potentially related to an insufficient CO elevation to compensate for the hypoxia-related loss in VO_2peak_ [18,19]. A sufficient CO increase was particularly prevented in individuals with higher (but normal) values of systemic BP (and BP changes from normoxia to hypoxia), likely due to enhanced baroreceptor activity and the associated reduction in sympathetic activation and cardiac output [20]. The reduced sympathetic activation may also explain why TPR was reduced in subjects showing the highest compensatory increase in CO. However, the fact that chemoreflex activation inhibits baroreflex activation and vice versa [21,22] complicates the interpretation of these results. Furthermore, when exercising with a small muscle mass in hypoxia (compared to normoxia), accumulation of metabolites (metaboreflex) in the contracting muscle (slightly increased La concentration in hypoxia) [23] and vascular tone regulation of the non-exercising musculature [14], influencing BP response, may interact in a complex manner. Moreover, central command, a feed-forward neural mechanism that transmits impulses to the motor neurons and in parallel modulates cardiovascular responses, may have played a role in exercise performance and CO adjustments [24]. 

In addition to CO limitations, muscle diffusion capacity may also affect single-leg exercise performance at altitude. We found an enhanced systemic O_2_ delivery and a lower a–vDO_2_ in moderate hypoxia, which point towards impaired oxygen extraction by the working muscles. The impairment could potentially result from the lower O_2_ pressure gradient and/or a shortened transit time [8,9,12]. Increased oxygen delivery to working muscles and lower a–vDO_2_ during steady-state single-leg [5,25,26], but also double-leg [10], knee-extension exercise has previously been demonstrated, albeit in more severe hypoxia than the present study. It needs to be acknowledged that in the present study, a–vDO_2_ was calculated and not measured, thus these data need to be interpreted with caution. 

### 4.1. Methodological Considerations and Limitations

Certainly, our preliminary observation of a relationship between systemic BP and CO (and thus also VO_2peak_) changes during exercise with a small muscle mass in acute moderate hypoxia must be interpreted with caution. As outlined, the relationship seems mainly driven by one subject and future studies involving a larger sample size are needed to confirm the present results. It also needs to be determined whether this relationship is valid under conditions of more severe hypoxia or when applying different exercise types. In this regard, it is important to mention that the type of exercise (e.g., one-legged dynamic knee extension vs. one-legged cycling), which also influences active muscle mass, may influence muscle blood flow and thus oxygen delivery. Consistent with this, it has been reported that the smaller the muscle mass (e.g., arms) and the less dynamic the exercise (e.g., intermittent isometric contraction), the more the muscular perfusion is impaired during maximal exercise [6,27]. The consequence is that maximal exercise is limited primarily by the intrinsic power of muscles rather than by O_2_ supply [6]. Since, in the present study, a one-legged cycle exercise was applied, deviating results compared with other studies may have resulted from differences in the type of exercise (e.g., knee extension). 

Sex differences in leg vasodilation during graded knee extensor exercise have been reported [28], which may have influenced the present results (males and females were included). Nonetheless, when analyzing changes from moderate altitude to sea level conditions, such differences should have little impact on the interpretation of the present data. Evidently, this only applies if there are little to no sex differences in responses to exercise in hypoxia. According to Shephard et al. [29], such differences should be minor during single-leg cycling and be most prominent when arm exercise is performed. In line with this, differences in mean VO_2peak_ and CO between altitude and sea level were similar between males and females in the present study (−2.9 mL/min/kg and 34.5 mL/min/kg for males and −2.8 mL/min/kg and 33.4 mL/min/kg for females, respectively).

The main limitation, which must certainly be acknowledged and may be considered critical, is the small sample size. Nonetheless, the uniform (in every participant) increases in CO and reductions in VO_2peak_ argue in favor of an existing compensatory increase in CO at moderate altitude, which was insufficient to restore normoxic VO_2peak_. Furthermore, we did not monitor direct changes of cardiovascular reflexes, but the observed association between MABP, CO, and VO_2peak_ suggest involvement of those reflexes, which have to be considered in future studies involving a larger sample size. 

### 4.2. Practical Considerations

Peak aerobic performance when exercising/working with a small muscle mass is acutely reduced even in moderate acute hypoxia. If performance is crucial, e.g., from a safety perspective, individual values of systemic BP and training involving a small muscle mass may be considered before going to altitude [30]. Additionally, acclimatization to altitude may be of importance for workers as well as athletes who have to perform with a small muscle mass (e.g., athletes with disabilities) at moderate altitude. With prolonged altitude exposure, neural adjustments change [31], which may contribute to the restoration of normoxic small muscle exercise performance, as demonstrated at higher altitudes [1]. In addition, it has been reported that acclimatization to moderate altitude may increase left ventricular muscle mass [32,33], potentially supporting compensatory increases in CO and thus performance involving a small muscle mass. Moreover, previous studies also demonstrated improved bilateral cycling performance after one-legged cycling training in normoxia [30] and hypoxia as well [34].

## 5. Conclusions

The present study found reduced P_peak_ and VO_2peak_, despite elevated systemic oxygen delivery, during one-legged cycling in acute, moderate (FiO_2_: 15%) hypoxia. The related CO increase was not sufficient to prevent the decline in P_peak_ and VO_2peak_. The magnitude of VO_2peak_ reduction was associated with individual CO responses, which again were correlated with individual MABP values. These observations likely indicate the involvement of baroreceptor activity in the explanation of CO and VO_2peak_ changes when exercising with a small muscle mass in acute moderate hypoxia. Not to be ignored, the enhanced systemic O_2_ delivery and the reduced a–vDO_2_ in moderate hypoxia may indicate a possible diffusion limitation. These novel, yet preliminary, findings may be of clinical/practical relevance and deserve further investigation. 

## Figures and Tables

**Table 1:** Baseline characteristics of the participants (*n* = 5).

Variables	Mean ± SD
Age (years)	42.6 ± 10.2
Weight (kg)	62.8 ± 10.6
Height (cm)	175.6 ± 5.7
BMI (kg/m^2^)	20.3 ± 2.5
HR_max_ (b/min)	180.6 ± 2.7
P_max_ (W/kg)	4.5 ± 0.7
VO_2max_ (ml/min/kg)	49.1 ± 7.0
La_max_ (mmol/l)	10.6 ± 1.6
Sport practice (h/week)	6.0 ± 0.8

Body mass index, BMI; maximal heart rate, HR_max_; maximal lactate concentration, La_max_; maximal power output, P_max_; maximal oxygen uptake, VO_2max_. Maximal values represent values during maximal two-legged cycling exercise.

**Table 2:** Resting (sitting on the cycle ergometer) values of the normoxia and hypoxia sessions.

Variables	Normoxia	Hypoxia	*p*-Value
HR (b/min)	59.4 ± 4.6	64.4 ± 4.6	0.03
MABP (mmHg)	92.2 ± 3.9	89.7 ± 6.6	0.43
SV (mL)	78.0 ± 5.8	73.6 ± 7.8	0.18
CO (L/min)	4.63 ± 0.46	4.72 ± 0.33	0.69
SpO_2_ (%)	96.5 ± 1.2	91.4 ± 1.1	0.04
CaO_2_ (mL/L)	190.2 ± 34.6	178.3 ± 25.3	0.14
Systemic O_2_ delivery (L/min)	0.88 ± 0.18	0.84 ± 0.15	0.35

Arterial oxygen content, CaO_2_; peripheral oxygen saturation, SpO_2_; cardiac output, CO; heart rate, HR; mean arterial blood pressure, MABP; stroke volume, SV; systemic O_2_ delivery = CO × CaO_2_.

**Table 3:** Peak one-legged cycling values during the normoxia and hypoxia sessions (*n* = 5).

Variables	Normoxia	Hypoxia	*p*-Value
HR_peak_ (b/min)	142.4 ± 6.9	155.6 ± 3.6	0.03
MABP (mmHg)	97.3 ± 10.4	99.2 ± 14.9	0.89
TPR (mmHg/L/min)	5.82 ± 1.34	5.21 ± 1.71	0.09
SV_peak_ (mL)	118.2 ± 21.4	121.8 ± 23.0	0.08
CO_peak_ (L/min)	16.83 ± 3.10	18.96 ± 3.59	0.04
paO_2_ (mmHg)	89.4 ± 3.2	76.8 ± 4.1	0.03
SpO_2_ (%)	95.9 ± 1.8	86.9 ± 1.1	0.03
Hb (g/dL)	15.2 ± 1.2	15.4 ± 1.8	0.35
CaO_2_ (mL/L)	197.9 ± 15.0	181.5 ± 19.3	0.04
a–vDO_2_ (mL/L)	137 ± 21	112 ± 19	0.03
Systemic O_2_ delivery (L/min)	3.37 ± 0.84	3.47 ± 0.89	0.04
P_peak_ (W)	154 ± 31	128 ± 26	0.03
P_peak_ (W/kg)	2.4 ± 0.2	2.0 ± 0.2	0.03
VO_2peak_ (mL/min)	2351 ± 719	2170 ± 706	0.04
VO_2peak_ (mL/min/kg)	36.8 ± 6.6	33.9 ± 6.9	0.04
La_peak_ (mmol/L)	8.2 ± 1.5	9.0 ± 1.6	0.04

Arterial oxygen content, CaO_2_ (calculated as: (Hb × 1.34 × SaO_2_) + (PaO_2_ × 0.003)); arterial–venous O_2_ difference, a–vDO_2_ (CaO_2_ − CvO_2_, calculated using the Fick equation a–vDO_2_ = VO_2_/CO); hemoglobin concentration, Hb; mean arterial blood pressure, MABP; peak cardiac output, CO_peak_; peak heart rate, HR_peak_; peak lactate concentration, La_peak_; peak oxygen uptake, VO_2peak_; peak power output, P_peak_; peak stroke volume, SV_peak_; peripheral oxygen saturation, SpO_2_; systemic O_2_ delivery = CO × CaO_2_.

## Data Availability

Data are contained within the article.
